# Obituary

**Published:** 1909-12-25

**Authors:** 


### Obituary.

The death is announced of Dr. John Wilton at the age of seventy-nine. He took his diploma in 1860, and became an M.D. of St. Andrews in 1873. He was a student at Guy's.

The death is announced of Dr. Joseph Cownley Brumwell, J.P., late of Burnley, aged eighty-six. Dr. Brumwell took his degree of M.D. at St. Andrews in 1862, having become an M.R.C.S. four years previously. He was the author of popular lectures on "The Chest: Its Construction, Uses, Accidents, and Disorders." He had retired from practice and made his home at Shanklin, I.W.

Mr. Henry Roscoe, recently appointed secretary and house governor to the North Staffordshire Infirmary, was found in his room at Stoke-on-Trent, on December 17, shot. Near the body was a revolver and two letters. He took his diploma in 1894. In 1898 he proceeded to South Africa, and afterwards held the position of district surgeon at Gwanda, Rhodesia. At the request of Mr. Cecil Rhodes he surveyed a site for Gwanda township, his suggestions being accepted and acted upon. He had also held the posts of honorary surgeon, Ancoats Hospital, Manchester, and resident medical officer, Crumpsall Infirmary, Manchester.

Mr. William James Russell, Ph.D., F.R.S., formerly lecturer in chemistry at St. Bartholomew's Hospital Medical School, died last month, aged seventy-nine. Dr. Russell received his scientific education at Heidelberg University, where he graduated as a Doctor of Philosophy nearly sixty years ago. Since he retired from the lectureship at St. Bartholomew's Hospital Dr. Russell published in *Nature* and elsewhere a series of articles upon the remarkable effect of contact of various articles with photographic plates in absolute darkness.

Dr. Alexander Groves Duff has died at Palmerston, New Zealand. He had a varied career during the Indian Mutiny. He was twice slightly wounded, and he received the medal and clasp. He was born at Calcutta and was seventy-five years old at the time of his death.

The death is announced of Dr. James Mackeand Cochrane, who was an M.D. of Toronto since 1884, when he was resident medical officer at the Toronto General Hospital. In 1890 be became Licentiate of the Royal College of Physicians. Previously, in 1885, he had held the post of resident physician to the City Hospital, Hamilton.

The death is announced of Mr. Marcus Gunn, at the age of fifty-nine. He took his degree of C.M. at Edinburgh in 1873, and became a Fellow of the Royal College of Surgeons in 1882. He also held at different periods the posts of senior surgeon to the Royal London Ophthalmic Hospital, Moorfields, ophthalmic surgeon to the National Hospital for the Paralysed and Epileptic, consulting surgeon to the West Ophthalmic Hospital. He was also President of the Ophthalmological Society, and at one time Arris and Gale lecturer to the Royal College of Surgeons, and ophthalmic surgeon to the Great Northern Central Hospital. He was also author of numerous publications.

The death is announced of Dr. Frederick William Blake, Deputy Inspector-General, R.N., at the age of eighty-five, who was Deputy-Inspector of Hospitals and Fleets. He was born in 1814. He received his medical education in London and qualified as a member of the Royal College of Surgeons in 1845, and became an M.D. of Aberdeen University in 1857. He entered the Royal Navy and served throughout the war with Russia, and was awarded the Crimean and Turkish medals. He was made a Fleet-Surgeon in 1870, and retired in 1879.

